# UK dementia prevention policies and initiatives across the life-course: a scoping review (2009–2024)

**DOI:** 10.1186/s12889-026-27289-1

**Published:** 2026-04-09

**Authors:** Naveena Luthra, AFM Saiful Islam

**Affiliations:** UCL Queen Square Institute of Neurology, 7 Queen Square, London, WC1N 3AR UK

**Keywords:** Dementia prevention, Brain health, Policy evaluation, Life-course, United Kingdom, Public health, Implementation science

## Abstract

**Background:**

Dementia is a growing public health challenge, with up to 45% of cases potentially preventable through modification of life-course risk factors. Although UK policies increasingly recognise prevention, the extent to which strategies are effectively implemented, evaluated, and equitably delivered remains unclear. This study aimed to systematically map and critically evaluate UK dementia-prevention strategies, with a focus on their reach, implementation, life-course coverage, and equity.

**Methods:**

A scoping review was conducted in accordance with PRISMA-ScR guidelines. We searched PubMed, Scopus, and Web of Science, alongside grey literature sources including government, NHS, and non-governmental organisation reports, to identify dementia-prevention strategies implemented in the UK between 2009 and 2024. Eligible records included national policies, public health campaigns, clinical initiatives, and professional resources addressing modifiable dementia risk factors. Strategies were analysed using the RE-AIM framework (Reach, Effectiveness, Adoption, Implementation, Maintenance), with additional consideration of life-course targeting and equity.

**Results:**

Twenty-three strategies were identified across policy, campaign, clinical, and professional domains. Dementia prevention was frequently embedded within broader non-communicable disease agendas rather than addressed through dedicated policies. Government-led campaigns (e.g. One You, Couch to 5 K) achieved high population reach but lacked explicit brain-health framing, while non-governmental campaigns (e.g. Reduce Your Risk, Think Brain Health) provided dementia-specific messaging but operated at smaller scale with limited evaluation. Clinical and professional initiatives (e.g. NICE NG16, NHS Health Checks) demonstrated variable uptake and inconsistent implementation. Across all domains, effectiveness and long-term maintenance were poorly evidenced due to limited follow-up and weak monitoring approaches. Most strategies focused on mid-life populations, with minimal attention to early- or late-life prevention, and no reporting on socioeconomic and ethnic inequalities.

**Conclusions:**

UK dementia-prevention efforts remain fragmented, under-evaluated, and insufficiently targeted across the life-course and many population groups. Strengthening prevention will require a dedicated national strategy with measurable indicators, integration of risk-reduction into routine healthcare and community settings, expansion of early-life interventions, and implementation of standardised evaluation frameworks with equity monitoring. Without these changes, opportunities to reduce dementia risk and its associated societal and economic burden may be missed.

**Supplementary Information:**

The online version contains supplementary material available at 10.1186/s12889-026-27289-1.

## Introduction

Dementia represents one of the greatest public health challenges of the 21st century. Globally, more than 55 million people are living with dementia, and prevalence is projected to almost triple by 2050, with associated costs exceeding US$1 trillion annually [1–3]. In the United Kingdom (UK), dementia is now the leading cause of death, affecting nearly one million people in 2024 and costing over £40 billion each year [4–6]. By 2040, prevalence is expected to reach 1.4 million, with national costs approaching £90 billion [4]. Despite advances in disease-modifying therapies, no curative treatment currently exists. Prevention through risk reduction therefore remains the most effective population-level strategy. The 2024 Lancet Commission estimated that up to 45% of dementia cases could be prevented or delayed by addressing 14 modifiable risk factors across the life-course [7], with tools such as the CAIDE Dementia Risk Score supporting midlife prevention efforts [8]. Throughout this review, “United Kingdom (UK)” refers to England, Scotland, Wales, and Northern Ireland. Where policies or initiatives apply only to England (e.g., NHS England programmes), this is stated explicitly. Interest in dementia prevention has grown within global frameworks, including the World Health Organisation’s Global Action Plan on Dementia [9] and successive Lancet Commission reports [1, 2, 7]. Since the publication of the UK’s National Dementia Strategy in 2009 [10], a range of initiatives have referenced prevention, including government policy commitments [11–14], national and non-governmental public health campaigns [15–17], clinical initiatives such as NHS Health Checks [18], and professional guidance and resources [19]. However, these efforts have developed in a piecemeal manner, are frequently embedded within broader non-communicable disease (NCD) agendas, and are rarely evaluated for reach, effectiveness, or sustainability [20–22].

International evidence highlights how more structured approaches to dementia prevention can be implemented. In Finland’s FINGER trial, combined approaches, including diet, physical activity, cognitive training, and vascular risk management, can reduce cognitive decline and have informed wider initiatives such as the World-Wide FINGERS network. Similarly, national and regional programmes in countries such as Australia have explored early-life dementia education, demonstrating improvements in knowledge and awareness. These approaches suggest that earlier, coordinated, and systematically evaluated interventions may strengthen long-term dementia prevention outcomes. In contrast, the UK has yet to implement a comparably integrated national prevention programme, indicating a gap between international evidence and domestic policy translation.

Despite an expanding evidence base, no previous synthesis has comprehensively mapped the UK’s dementia-prevention landscape. Earlier reviews have focused on specific interventions or risk factors [23–25], but none have examined how policy, campaign, clinical, and professional domains interact to influence implementation and equity. A system-level understanding is essential for translating modifiable risk factor evidence into actionable national strategies, a priority for clinicians, researchers, and policymakers. Scoping reviews are particularly suited to this purpose: unlike systematic reviews, which address narrowly defined intervention effects, scoping reviews capture the breadth and heterogeneity of evidence, including grey literature and policy documents. This enables mapping of activity, identification of gaps, and evaluation of strategic coherence across levels of prevention.

Accordingly, this review aimed to provide the first comprehensive synthesis of UK dementia-prevention policies and initiatives implemented between 2009 and 2024, representing the period from the introduction of the first National Dementia Strategy to the most recent evidence available at the time of the review. Guided by PRISMA-ScR [26], the review appraised strategies using an implementation framework to assess their reach, effectiveness, adoption, implementation, and maintenance [27]. The objectives were to:

1. Describe the scope and characteristics of UK dementia-prevention strategies across policy, campaign, clinical, and professional domains;
2. Evaluate their strengths, weaknesses, and gaps; and
3. Identify cross-cutting priorities to inform future policy and translational dementia-prevention efforts in the UK and internationally.

In this review, *dementia prevention* refers to population and policy-level actions that lower incidence at scale, whereas *risk reduction* denotes individual and service-level measures (e.g., vascular risk control, smoking cessation, hearing correction) delivered across the life-course.

## Methods

### Study design

We conducted a scoping review to map the breadth and characteristics of heterogeneous evidence on dementia-prevention strategies rather than to assess intervention effectiveness. Scoping reviews are suited to this topic because they enable inclusion of diverse study types, policy documents, and grey literature, and allow examination of gaps across a wide range of initiatives. Reporting followed the PRISMA extension for Scoping Reviews (PRISMA-ScR) [26]. Dementia prevention was defined broadly to include both population and service-level strategies.

### Open-science deposit

An earlier version of this manuscript, including detailed methods and protocol information, was deposited on the Open Science Framework [28] to ensure transparency and accessibility of materials.

### Search strategy

We searched for dementia-prevention strategies published in the United Kingdom between January 2009 (the launch of the first National Dementia Strategy [12]) and April 2024. All searches were conducted in April 2024. Peer-reviewed literature was searched in PubMed, Scopus, and Web of Science using Boolean combinations of “dementia prevention,” “risk reduction,” “policy,” “campaign,” “initiative,” “NHS,” and “United Kingdom.” Grey-literature sources were identified through targeted searches of UK government portals (Department of Health and Social Care, NHS England, NICE), non-governmental organisations (Alzheimer’s Society, Alzheimer’s Research UK), and professional bodies (Public Health England/Office for Health Improvement and Disparities, Royal Colleges). Reference lists of included documents were screened for additional items. Because health policy is devolved across the UK, grey literature searches also included portals for the Scottish Government, the Welsh Government, NHS Scotland, and the Northern Ireland Department of Health. Few dementia-prevention-specific documents were identified in these sources, and most available strategies were UK-wide or England-led, as reflected in the final evidence base.

### Eligibility criteria

Documents were included if they:

1. Described a dementia-prevention or risk-reduction strategy implemented in the UK (January 2009–April 2024); both UK-wide strategies and nation-specific initiatives from England, Scotland, Wales, or Northern Ireland were eligible for inclusion.
2. Addressed at least one domain: national or regional policies, public-health campaigns, clinical initiatives (including NHS Health Checks and NICE guidance), or professional resources;
3. Focused on population- or service-level prevention or risk-reduction measures (e.g., lifestyle modification, vascular-risk management, hearing correction, smoking cessation, or physical-activity promotion); and
4. Available in English.

Exclusion criteria included documents focused solely on dementia care, diagnosis, or treatment; primary biomedical or pharmaceutical research without a prevention component; international documents lacking UK implementation details; and duplicate or media reports without substantive information.

### Study selection

**Fig. 1:**
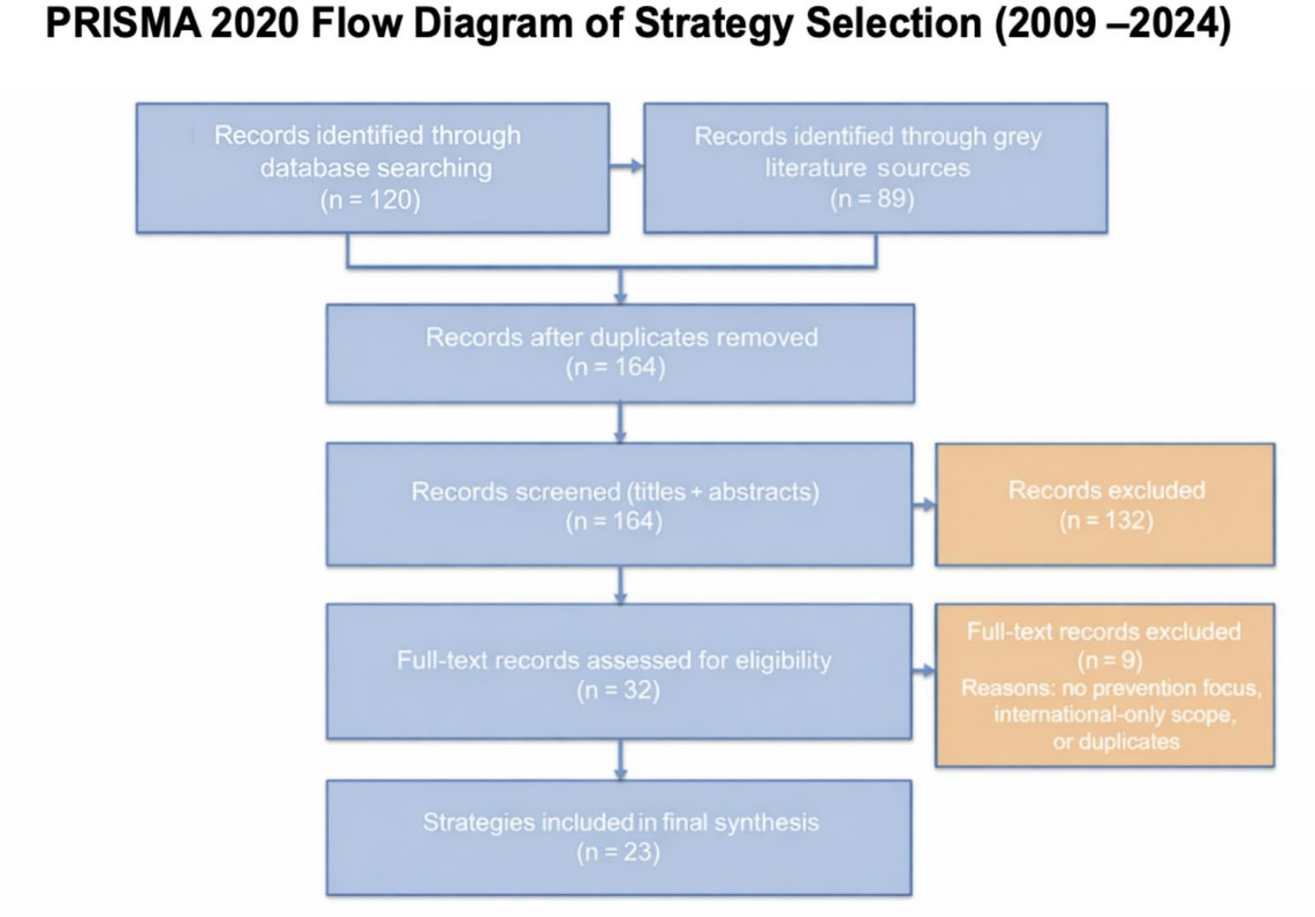
(PRISMA 2020 Flow Diagram): Records identified through database searching (*n* = 120) and grey literature sources (*n* = 89), total (*n* = 209); records after duplicates removed (*n* = 164); records screened (titles and abstracts) (*n* = 164); full-text records assessed for eligibility (*n* = 32); strategies included (*n* = 23). Exclusions of full-text records (*n* = 9) were due to the absence of a prevention component, international-only scope, or duplication

Two reviewers independently screened all titles, abstracts, and full texts against the eligibility criteria. Any disagreements were resolved through discussion and consensus between the two reviewers; no third reviewer was required. The selection process is summarised in a PRISMA flow diagram (Fig. 1). Although records/documents were screened for eligibility, the unit of analysis was defined as ‘strategies’, as each included document described a distinct dementia-prevention initiative. Of the 209 records identified, 23 records describing dementia-prevention strategies met the inclusion criteria.

### Data charting

Data were extracted independently by two reviewers using a structured Microsoft Excel template. Extracted variables included publication year, source, sponsoring organisation, strategy type (policy, campaign, clinical initiative, professional resource), target population, risk factors addressed, delivery mechanism, implementation approach, monitoring and evaluation methods, and equity considerations. Extracted data were cross-checked for consistency, and any discrepancies were resolved through discussion and consensus.

### Grey-literature appraisal

Because many included strategies derived from government or non-governmental organisations (NGOs) sources, grey-literature materials were appraised for authoritativeness, accuracy, currency, and transparency using adapted AACODS criteria (Authority, Accuracy, Coverage, Objectivity, Date, Significance). Only materials meeting these criteria were included in the synthesis.

### Synthesis and visualisation

Extracted data were summarised descriptively and synthesised narratively in accordance with PRISMA-ScR guidance [26]. Strategies were grouped by domain (policy, campaign, clinical, professional), and cross-cutting themes were identified with attention to equity and sustainability. Visualisations were created in Microsoft Excel and refined in Adobe Illustrator to generate the RE-AIM (Reach, Effectiveness, Adoption, Implementation, Maintenance) radar chart and life-course map.

For each RE-AIM dimension, evidence was assessed qualitatively based on the information reported in the included records. Ratings such as high, moderate, weak, low, or absent reflected the extent, specificity, and continuity of reported evidence rather than formal effect-size estimation. “High” indicated explicit and substantial evidence of delivery or evaluation; “moderate” indicated partial but relevant evidence; “weak” or “low” indicated limited or indirect evidence; and “absent” indicated that no relevant evidence was reported. Ratings were assigned by reviewer consensus and were intended to support comparative interpretation across heterogeneous strategies.

### Frameworks applied

The RE-AIM framework [27] served as the primary analytic lens for assessing breadth and depth across the five dimensions (Reach, Effectiveness, Adoption, Implementation, Maintenance). This framework was selected due to its suitability for evaluating the implementation and public health impact of complex interventions across multiple domains. Interpretation was informed by complementary behavioural and socioecological models, specifically the Capability, Opportunity, Motivation–Behaviour (COM-B) model [29] and the Health Belief Model [30], which provided insight into individual, organisational, and structural determinants of prevention. The COM-B model was used to understand behavioural drivers of risk-reduction strategies, while the Health Belief Model provided insight into how individual perceptions may influence engagement with prevention initiatives. Together, these frameworks enabled a multi-level interpretation of implementation and behavioural factors.

### Patient and public involvement

Patients and members of the public were not involved in the design, conduct, or reporting of this review, as the study was based solely on analysis of publicly available documents and did not involve primary data collection.

### Ethical considerations

Ethical approval was not required, as all data sources were publicly available policy and strategy documents.

## Results

### Study selection and characteristics

A total of 209 records were identified, of which 45 duplicates were removed, leaving 164 for title and abstract screening. Thirty-two full-text records were assessed for eligibility, and 23 included records describing dementia-prevention strategies were retained for synthesis. Although records/documents were screened for inclusion, the unit of analysis was the strategy described within each included record. The selection process is summarised in the PRISMA 2020 flow diagram (Fig. 1).

The 23 included strategies comprised 7 policy documents, 5 public-health campaigns, 4 clinical initiatives, and 7 professional resources or guidance documents. Most were national in scope or intended for UK-wide or England-wide implementation, while only a small number reflected regional or pilot-level activity. Publication activity increased after 2015, with most included strategies published between 2016 and 2024. All included strategies are detailed in Supplementary Table S1.

Not all included records reported evidence across all five RE-AIM dimensions; reach and adoption were more commonly described than effectiveness, implementation quality, maintenance, or long-term outcomes.

### Policy and campaigns

National policy documents, including the *Prime Minister’s Challenges* (2015, 2020), the *NHS Long Term Plan* [14], the *Major Conditions Strategy* (2023) [11], and the *WHO Global Action Plan* (2017–25) [9], acknowledged modifiable risk factors but rarely translated them into measurable dementia-specific outcomes (Table 1).

Although each devolved nation (Scotland, Wales, Northern Ireland) has its own dementia strategy, these documents primarily focus on diagnosis, post-diagnostic support, and care pathways rather than prevention. Because they did not include dementia-specific prevention actions or risk-reduction initiatives, they did not meet the inclusion criteria for this review. For this reason, the evidence base is dominated by UK-wide and England-led strategies.

**Fig. 2:**
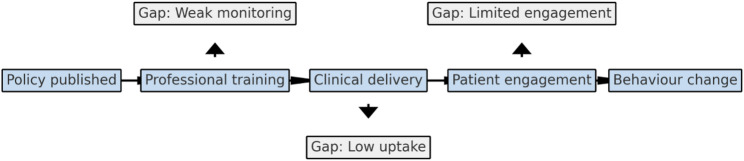
Policy-to-Outcome Gaps in UK Dementia Prevention Strategies. High-level commitments are seldom translated into measurable implementation or monitoring indicators

*The Dementia 2020 Challenge: Implementation Plan* actioned presented much on dementia awareness however, included few explicit dementia prevention indicators, limiting the monitoring of implementation [31]. Across all, reach, and adoption were moderate; however, effectiveness and maintenance remained weak. Professional resources including Health Matters Approaches to Reduce Dementia Risk provides evidence summaries and implementation guidance for practitioners, public visibility and outcome evaluation were limited [32]. Figure 2 illustrates the persistent policy-to-outcome gap between high-level policy commitments and the absence of concrete monitoring or outcome indicators.

**Table 1:** *RE-AIM* evaluation of UK national dementia policies (2009–2024) : Policies showed partial adoption but minimal maintenance; none incorporated dementia-specific prevention metrics

PM’s Challenge (2015) [33]	Moderate	Weak	Partial	Uneven delivery	Absent
PM’s Challenge (2020) [33]	Moderate	Weak	Partial	Variable	Absent
NHS Long Term Plan [7, 14]	Moderate	Weak (no dementia-specific prevention metrics)	Adopted nationally	Variable across ICSs	Absent
Major Conditions Strategy (2023) [11]	Low	Weak	Adopted	Minimal	Absent
WHO Global Action Plan (2017–25) [9]	Moderate	Weak	Adopted	Variable	Absent

**Fig. 3:**
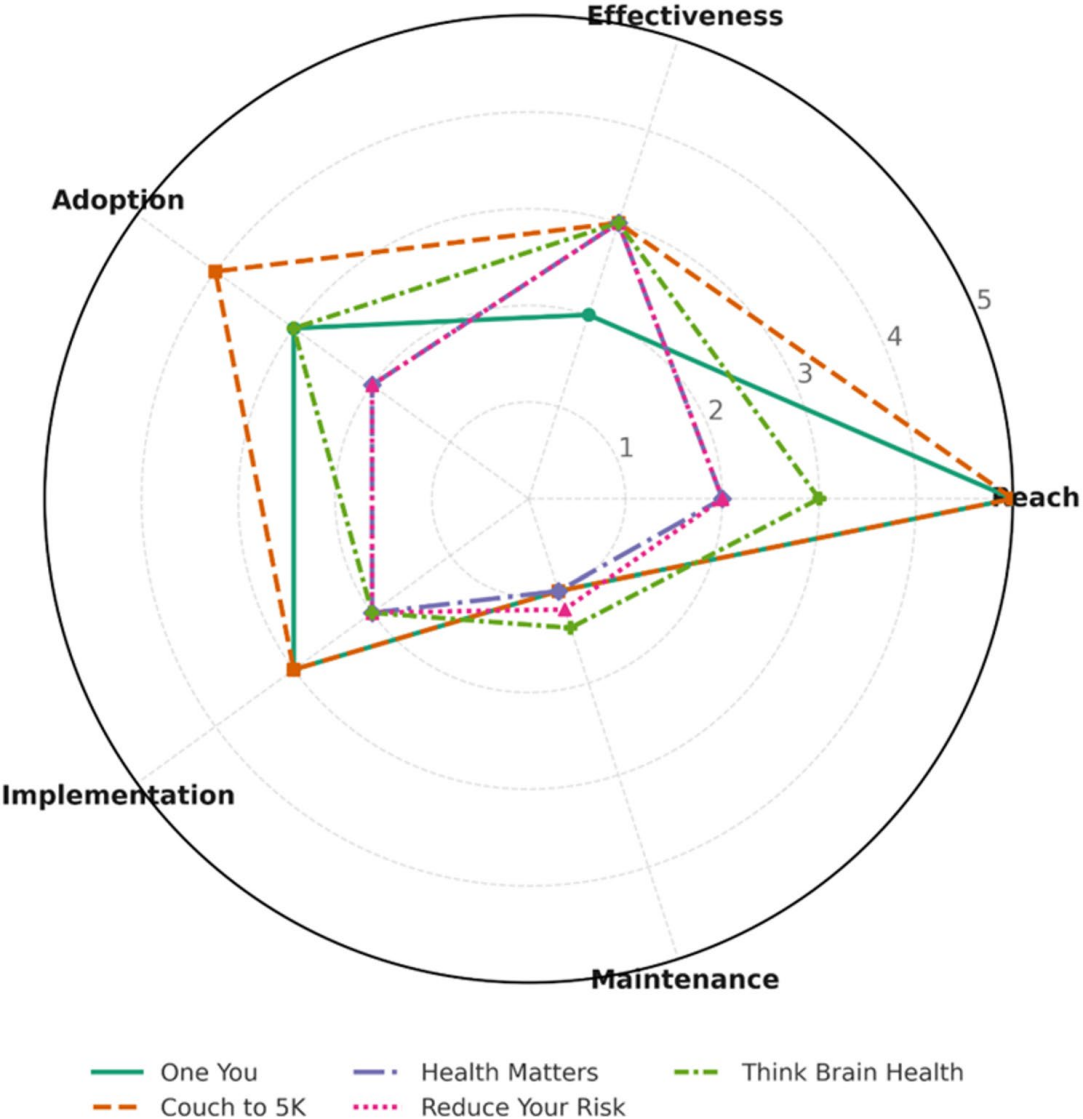
RE-AIM Radar Chart for UK Dementia Prevention Campaigns. Comparative visualisation of five major campaigns across RE-AIM dimensions (Reach, Effectiveness, Adoption, Implementation, Maintenance). Government initiatives achieved broader reach; NGO campaigns scored higher on specificity but lower on sustainability

Public-health campaigns demonstrated a trade-off between scale and specificity (Table 2; Fig. 3). Table 2 shows a consistent scale, specificity trade-off: government-led campaigns achieved the widest reach but seldom framed lifestyle modification as dementia prevention, whereas dementia-specific NGO campaigns offered clearer brain-health messaging but lacked comparable scale, continuity, and outcome evaluation. Public attitudes research indicates that awareness of dementia prevention remains limited as adults fail to understand the role of lifestyle and vascular risk factors in later-life dementia risk [34]. Government-led initiatives such as *One You* and *Couch to 5 K* achieved wide population reach but seldom framed messages in terms of brain health. In contrast, NGO-led campaigns such as *Reduce Your Risk* [16] and *Think Brain Health* [15, 17] provided explicit dementia-prevention messaging but remained smaller in scale, short-lived, and rarely evaluated. Across campaigns, reach scored highest on RE-AIM, while effectiveness and maintenance were weakest.

**Table 2:** Reach vs. specificity of UK dementia prevention campaigns. Government-led programmes achieved wide reach but lacked brain-health framing; NGO campaigns provided targeted messaging but limited scale and follow-up

Campaign	Lead Organisation	Reach	Dementia-specific?	Key Outcome/Gap
One You	PHE	High	No	No dementia-specific data
Couch to 5 K	NHS/PHE/Sport England	High	No	No cognitive health link
Health Matters	Public Health England	Moderate	Partial	Not accessible to the public
Reduce Your Risk	Alzheimer’s Research UK	Low–Moderate	Yes	Small-scale, no follow-up
Think Brain Health	Alzheimer’s Research UK	Moderate	Yes	High engagement, no outcome data

### Clinical and professional practice

Clinical and professional strategies demonstrate uneven adoption and limited sustainability. NICE guidance NG16 [12] recommended mid-life vascular-risk management, physical activity, and smoking cessation, yet implementation remained voluntary. *NHS Health Check* pilots incorporating dementia-risk discussions [21, 29] increased short-term awareness but provided little evidence of sustained behaviour change as documentation of dementia-specific risk discussions remains inconsistent with routine delivery [35]. Professional resources such as *All Our Health* and OHID toolkits [19] were widely available but optional, and uptake was not systematically monitored. Systematic review results suggest that effective treatment of vascular risk factors including hypertension, may reduce dementia incidence, highlighting the rationale for midlife prevention strategies [36].

The NHS England Dementia Programme Update [18] focused primarily on diagnosis recovery rather than structured prevention delivery. Collectively, clinical and professional initiatives demonstrated partial adoption and weak maintenance across RE-AIM dimensions.

### Life-course perspective

**Fig. 4:**
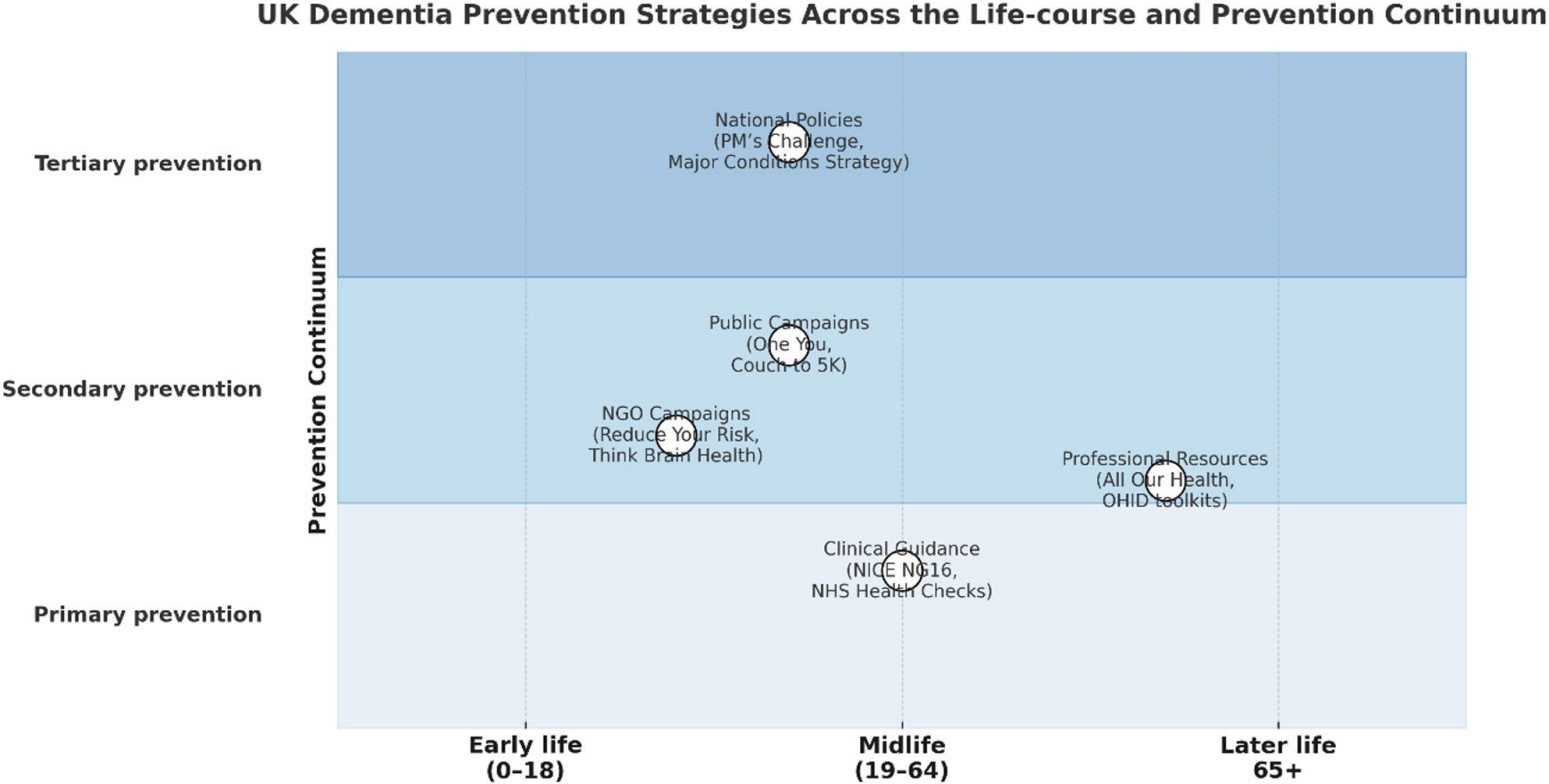
Life-course mapping of UK dementia prevention strategies. Strategies concentrate on mid-life; early- and late-life stages remain sparsely addressed, highlighting missed opportunities for comprehensive prevention

Mapping of prevention strategies across the life-course revealed a marked imbalance in both timing and focus (Fig. 4). Most UK initiatives cluster in mid-life and emphasise primary prevention through lifestyle change. National policies (e.g., *Prime Minister’s Challenge*, *Major Conditions Strategy*) and government campaigns (*One You*, *Couch to 5 K*) primarily targeted adults aged 40–64. Dementia-specific NGO campaigns offered sharper brain-health framing but were small in scale and temporary. Clinical initiatives (NICE NG16, NHS Health Check pilots) addressed mid-life vascular risk yet remained voluntary. Very few strategies targeted early-life determinants, and almost none addressed tertiary prevention after diagnosis.

### Socioeconomic and structural inequalities

Socioeconomic inequalities significantly influence dementia risk, diagnosis, and prevention. Evidence indicates that wealth-related disparities account for a substantial proportion of dementia risk, with approximately half of this difference mediated by modifiable lifestyle factors such as physical activity, diet, smoking, and cardiovascular health [20]. Although interventions targeting these factors, including cardiovascular risk management, exercise, healthy diet, and lifelong learning, are effective in reducing cognitive decline, access remains uneven. Disadvantaged communities face a higher prevalence of risk factors and fewer opportunities for engagement [21]. In the UK, diagnosis rates vary by region, ethnicity, and socioeconomic status. Rural or deprived areas often experience limited dementia-friendly services, while cultural and language barriers hinder uptake among minority ethnic groups. Global evidence presents delays and inequalities in dementia diagnosis, may further limit secondary or tertiary prevention, this is seen in disadvantaged populations [37]. Despite strategic commitments, prevention remains underdeveloped, with current frameworks prioritising early diagnosis and care rather than addressing upstream social determinants of dementia [22]. This gap represents a critical weakness in the *Reach* and *Adoption* dimensions of RE-AIM.

Few included strategies explicitly reported the use of behavioural theory in their original design or implementation. In most cases, behavioural models such as COM-B and the Health Belief Model were applied in this review to aid interpretation rather than being embedded within the source strategies themselves. Similarly, explicit patient or public co-development was rarely reported in the included records. Although some strategies acknowledged inequalities or targeted broad population, risk factors associated with deprivation, ethnicity, or access to care, equity considerations were usually limited to general policy statements rather than operationalised outreach, tailored delivery, or stratified outcome reporting.

### Gaps across UK dementia-prevention domains

Understanding evidence across policy, campaign, clinical and equity domains revealed several persistent structural and implementation gaps (Table 3).Prevention remains fragmented and inconsistently delivered. Large-scale government campaigns achieve population reach but lack explicit brain-health framing, while dementia-specific NGO efforts are smaller and rarely evaluated. Clinical guidance and NHS Health Check pilots show partial uptake and limited sustainability. Prevention activities remained concentrated in midlife despite stronger evidence for early-life interventions. Equity considerations are sparse, and evaluation frameworks seldom include long-term or equity-stratified monitoring.

Together, these gaps convey the need for a dedicated national dementia-prevention policy, integrated monitoring, and equity-focused outreach.

**Table 3:** Gaps Across the UK Dementia-Prevention Landscape

Domain	Gap Identified
Policy	No dedicated dementia prevention policy; prevention diluted into broader NCD agendas
Campaigns	Large-scale government campaigns achieve reach but lack brain health framing; NGO campaigns provide dementia-specific messaging but remain small and under-evaluated
Clinical Practice	NICE guidance and NHS Health Check pilots are voluntary; adoption is uneven and not sustained
Life-course Focus	Prevention efforts concentrated in late life despite stronger evidence for early- and midlife interventions
Equity	Limited tailoring for deprived and minority populations; risk of widening health inequalities
Evaluation & Monitoring	Almost no long-term monitoring, minimal outcome reporting, and absence of equity-stratified indicators

### Proposed framework for UK dementia-prevention strategies

To strengthen accountability and overcome the deficiencies outlined in Table 3, a structured monitoring approach is essential and should be implemented soon. As shown in Table 3, the most persistent cross-domain weaknesses were the absence of a dedicated prevention policy, inconsistent implementation in routine care, and limited equity-sensitive monitoring. Table 4 presents a proposed minimal monitoring framework designed to operationalise measurable indicators across key implementation dimensions. Table 4 is intended as a pragmatic response to the evidence gaps identified in this review, particularly the lack of standardised indicators for reach, implementation, maintenance, and equity. By incorporating equity stratification and sustainability metrics, this framework offers a coherent solution for embedding evaluation into UK dementia-prevention policy and practice, ensuring that future strategies are coherent, measurable, and equitable. This framework defines a small, pragmatic indicator set for national accountability, including process measures (e.g., number of active dementia-prevention campaigns), reach (population exposure or service uptake), implementation (adoption of NICE NG16 guidance), outcome proxies (smoking prevalence, controlled hypertension, hearing checks, physical activity levels), and maintenance (programmed continuation ≥ 2 years). All indicators are to be stratified by deprivation and ethnicity to ensure equity monitoring.

**Table 4:** Proposed Minimal Monitoring Set for UK Dementia-Prevention Strategies

RE-AIM Dimension	Example Indicators	Notes
Process	Number of dementia-specific campaigns launched annually; number of NHS regions with an active dementia prevention plan	Captures structural commitment and investment
Reach	% of target population exposed to campaign messaging; % uptake of NHS Health Checks including dementia risk component	Must be reported with equity stratification
Implementation	% of GPs delivering dementia risk-reduction counselling; proportion of regions applying NICE NG16 guidance	Tracks adoption consistency across NHS settings
Effectiveness (proxy outcomes)	Midlife smoking prevalence; proportion with controlled hypertension; % completing hearing checks; physical activity levels	Uses routinely collected NHS or national survey data
Maintenance	Proportion of programmes evaluated beyond 2 years; retention of dementia prevention content in NHS Health Checks; continued campaign funding	Addresses sustainability over time
Equity (cross-cutting)	All indicators above stratified by deprivation quintile and ethnicity	Ensures inequalities are monitored and addressed

### International comparisons

Although UK strategies reference international exemplars, few insights have been operationalised. The Finnish *FINGER* trial demonstrated that multidomain interventions; diet, exercise, cognitive training, vascular-risk management, reduced cognitive decline [23, 24]. The *World-Wide FINGERS* network [25] confirmed the feasibility of scaling such approaches internationally. International monitoring reports present uneven progress in implementation based on the WHO Global Dementia Action Plan, with prevention targets among the least consistently achieved [38]. Despite these models, the UK has not implemented a comparable national programme. The contrast underscores limited translation of global evidence into domestic policy and weak alignment with the WHO Global Action Plan [9].

### Interpretation within policy and implementation context

Despite consistent policy rhetoric since the 2009 *National Dementia Strategy* [10], the UK has yet to establish an integrated national dementia-prevention framework. Most policy documents embed dementia within broader non-communicable disease agendas [11–14], diluting disease-specific visibility and accountability. While public-health campaigns such as *One You* and *Couch to 5 K* achieved high population reach, their non-specific framing limited their translational impact on dementia risk awareness. NGO-led campaigns such as *Think Brain Health* [15, 17] and *Reduce Your Risk* [16] adopted targeted messaging but lacked scale and continuity. These patterns highlight a recurring scale–specificity imbalance: large programmes achieve reach without depth, while dementia-specific efforts achieve focus without longevity. Challenges are seen in translating dementia risk prediction models into routine clinical practice, including the uncertainty about long-term impact [39].

Clinical and professional initiatives exhibited these inconsistencies. NICE guidance NG16 [12] and NHS Health Check pilots [21, 29] provided clear opportunities to embed dementia-risk reduction in routine care, yet implementation remained voluntary. This optionality also limits sustainability, aligning with RE-AIM findings that organisational structures and mandates strongly influence long-term implementation. Professional training resources, including All Our Health and OHID toolkits [19], remain underused due to the absence of monitoring mechanisms or incentives.

### Behavioural and socioecological insights

Behavioural and socioecological frameworks help explain why existing initiatives often fail to translate into meaningful and sustained behaviour change. According to the COM-B (Capability, Opportunity, Motivation – Behaviour) model [29], capability and motivation were addressed through education and awareness campaigns, but opportunity, the environmental and structural support for sustained behaviour change, was insufficient. The Health Belief Model [30] suggests that while perceived susceptibility and benefits were communicated effectively, cues to action and enabling conditions were rarely reinforced. From a socioecological perspective [40], most strategies operated at the individual level, with limited engagement of community, institutional, or policy environments. This imbalance undermines long-term maintenance, explaining why even well-received campaigns have not translated into measurable reductions in dementia risk at the population scale.

### Equity and inclusion

Equity remains the weakest dimension across all prevention domains. Few initiatives reported outcomes stratified by socioeconomic deprivation or ethnicity, despite substantial evidence of unequal dementia burden [20–22]. Limited reach in disadvantaged communities and underrepresentation of ethnic minorities reflect structural barriers within mainstream prevention delivery. To address this gap, this review developed a minimal national monitoring set (Table 4) incorporating equity indicators—such as stratification by deprivation quintile and ethnicity, within each RE-AIM domain. Embedding these indicators into NHS reporting systems would allow systematic assessment of reach and uptake, ensuring that prevention benefits are distributed equitably.

However, this review has several limitations. First, although grey-literature searches included devolved-nation sources, few dementia-prevention-specific documents were identified from Scotland, Wales, or Northern Ireland. As a result, the evidence base is dominated by UK-wide and England-led strategies. Second, the scarcity of peer-reviewed evaluations of dementia-prevention initiatives meant that much of the included material derived from policy and organisational documents, which vary in detail and methodological rigour. Third, because many strategies lacked measurable outcomes or equity-stratified reporting, assessment of effectiveness and long-term impact was limited. These constraints reflect broader gaps in the UK dementia-prevention evidence base and highlight the need for more systematic evaluation and transparent monitoring.

### Implications for translational research and policy reform

The findings have clear implications for translational dementia research, service delivery, and policy development. Embedding dementia-risk discussions into NHS Health Checks represents a scalable translational model linking public-health policy with clinical prevention. Embedding measurable brain-health indicators into electronic health records could enable ongoing evaluation of risk-reduction counselling, medication adherence, and vascular control, bridging population-level strategy and patient-level outcomes. For research, the review highlights opportunities to test implementation frameworks (e.g., RE-AIM, COM-B) in real-world dementia-prevention programmes, advancing the evidence base for effective translation from behavioural theory to practice.

More broadly, the review presents the need for a cohesive UK dementia-prevention strategy, supported by long-term investment, equitable reach, and robust monitoring. Priorities for reform include national policy integration, standardised primary-care delivery of risk reduction, expanded life-course targeting, and sustained equity-focused outreach. These priorities provide a foundation for developing a coordinated, measurable, equitable prevention system capable of reducing future dementia incidence.

### Recommendation and policy priorities

Across 15 years of policy and practice, UK dementia-prevention efforts have expanded in number but not in integration or sustainability. Maintenance, equity, and accountability remain weakest across RE-AIM domains. Six cross-cutting priorities emerge from this review and are summarised below:

1. Establish a dedicated national dementia-prevention policy with defined metrics.
2. Adopt explicit brain-health framing across all campaigns.
3. Integrate risk-reduction counselling as a standard primary-care function.
4. Target earlier life stages through vascular and lifestyle interventions.
5. Prioritise equity-focused outreach to disadvantaged and minority communities; and.
6. Implement a minimal monitoring set (Table 4) within NHS reporting for accountability.

Implementing these priorities would transform the UK’s fragmented efforts into a coordinated, measurable, and equitable dementia-prevention system. Such integration would also create a foundation for translational research, bridging behavioural evidence, clinical application, and population-level impact.

## Discussion

This scoping review provides the first broad synthesis of UK dementia-prevention activity from 2009 to 2024, identifying 23 strategies across policy, public health, clinical initiatives, and professional resources. These demonstrate increasing attention to risk reduction but limited coherence or accountability, with the UK presenting strong policy rhetoric but weaker implementation data compared with peer nations [41]. Most interventions focused on mid-life lifestyle modification, with early-life and post-diagnosis stages rarely addressed. RE-AIM analysis showed moderate reach and adoption, but weak effectiveness, implementation, and particularly maintenance, indicating continued fragmentation and largely short-term approaches.

The review’s findings are consistent with international evidence demonstrating a gap between policy commitments and implementation of dementia prevention strategies. Multidomain interventions, such as those evaluated in the Finnish FINGER trial, have shown that coordinated approaches combining lifestyle modification, vascular risk management, and cognitive training can reduce cognitive decline. However, similar large-scale, structured prevention programmes have not been implemented nationally within the UK. This reflects a broader pattern identified in global dementia policy reviews, where prevention is recognised at a strategic level but fail to be translated into measurable, sustained public health action. These findings can be further understood through behavioural and implementation models. According to the COM-B model, many strategies addressed capability and motivation through awareness and education but insufficiently create better opportunity through structural or environmental support. Similarly, the Health Belief Model suggests that while perceived risk and benefits were communicated, cues to action and sustained reinforcement were limited. From an implementation perspective, RE-AIM analysis demonstrated that reach was often achieved, but effectiveness, implementation consistency, and maintenance were weak, reinforcing the need for system-level integration and long-term monitoring.

The observed fragmentation of UK dementia-prevention strategies may be explained by structural and implementation challenges common to complex public health interventions. While policies and campaigns address behavioural risk factors, they often lack integration across healthcare systems and community settings, limiting long-term impact. The imbalance between scale and specificity identified in this review, where government programmes achieve broad reach but lack dementia-specific framing, and NGO initiatives provide targeted messaging but limited scale, highlights a major disconnect between policy design and population-level behaviour change.

Strengths include systematic mapping across the UK over 15 years; inclusion of national policies, campaigns, clinical initiatives, and professional resources; and grey literature that captures real-world practice. Application of multiple behavioural and implementation frameworks (RE-AIM, COM-B [29], Health Belief Model [30], socioecological models [40]) enhanced interpretation, while double screening, duplicate extraction, and open-science protocol sharing [28] strengthened transparency and rigour.

These limitations have important implications for interpretation. The reliance on grey literature and policy documents reflects real-world practice but limits the ability to assess effectiveness using standardised outcome measures. The absence of consistent evaluation data across strategies also restricts comparison and may underestimate or overestimate their true impact. Limitations also include restriction to English-language UK sources, potential omission of non-indexed or regional initiatives, and reliance on AACODS for quality assessment, which evaluates credibility rather than methodological strength. Notably, few peer-reviewed studies evaluating UK dementia-prevention strategies were identified, highlighting a gap between policy development and academic evaluation. As a scoping review, the purpose was mapping rather than effect estimation, and limited reporting on long-term outcomes or equity-constrained synthesis.

Overall, dementia-prevention efforts in the UK remain fragmented, uneven across life stages and population groups, and insufficiently evaluated. The minimal monitoring framework and six cross-cutting priorities proposed here offer a pathway to coordinated national action; embedding measurable prevention indicators within NHS and public-health systems could improve accountability and accelerate population-level risk reduction.

## Supplementary Information

Supplementary Material 1.

## Data Availability

All data used in this review were derived from publicly available policy documents, organisational reports, and published literature. The datasets generated and analysed during the current study consist solely of these publicly accessible sources and are fully cited within the manuscript. No additional datasets were created.

## Abbreviations

AACODS: Authority, Accuracy, Coverage, Objectivity, Date, Significance
ARUK: Alzheimer’s Research UK
CAIDE: Cardiovascular Risk Factors, Aging and Dementia (Risk Score)
COM B: Capability, Opportunity, Motivation – Behaviour
DHSC: Department of Health and Social Care
FINGER: Finnish Geriatric Intervention Study to Prevent Cognitive Impairment and Disability
GP: General Practitioner
ICS: Integrated Care System
NCD: Non Communicable Disease
NGO: Non Governmental Organisation
NHS: National Health Service
NICE: National Institute for Health and Care Excellence
OHID: Office for Health Improvement and Disparities
PHE: Public Health England
PRISMA ScR: Preferred Reporting Items for Systematic Reviews and Meta Analyses – Scoping Review extension
RE AIM: Reach, Effectiveness, Adoption, Implementation, Maintenance
UK: United Kingdom
WHO: World Health Organisation
WW FINGERS: World Wide FINGERS Network
