# Supplementary material for: UK dementia prevention policies and initiatives across the life-course: a scoping review (2009–2024)
Source: BMC Public Health. 2026 Apr 9;26:1616. doi: 10.1186/s12889-026-27289-1 (PMC13191920; doi:10.1186/s12889-026-27289-1)
Supplement: Supplementary file 1 — Supplementary Material 1. [file 12889_2026_27289_MOESM1_ESM.docx]

**Supplementary Table S1. UK dementia prevention strategies (2009–2024)**

| No | Strategy / Programme | Year(s) | Lead organisation | Target population | Risk factors addressed | Delivery mechanism | Evaluation/findings |
| --- | --- | --- | --- | --- | --- | --- | --- |
| 1 | Living Well with Dementia: A National Dementia Strategy | 2009→ | Department of Health | Whole population/system | Awareness; earlier risk discussion | National strategy & guidance | Framed prevention within broader dementia policy; limited prevention metrics |
| 2 | Prime Minister’s Challenge on Dementia 2020 | 2015 | DH/DHSC | Whole population | Broad lifestyle & system actions | Cross-government programme | Moderate reach; weak prevention outcomes; limited maintenance (RE-AIM) |
| 3 | Dementia 2020 Challenge – Implementation Plan | 2016 | DHSC | System / NHS | Embedding risk messaging in practice | Implementation plan | Delivery varied; few dementia-specific prevention indicators |
| 4 | WHO Global Action Plan on Dementia (UK adoption) | 2017–2025 | WHO / UK Govt | Member-state level | National indicators incl. risk reduction | International framework | Adoption noted; UK reporting weak vs indicators |
| 5 | NHS Long Term Plan (dementia elements) | 2019→ | NHS England | NHS/system | Prevention rhetoric; midlife focus implied | National plan | No dementia-specific prevention metrics; variable implementation |
| 6 | Major Conditions Strategy – Case for Change | 2023 | DHSC | Cross-condition | Vascular risk, lifestyle, inequalities | Policy framework | Prevention framed cross-condition; few dementia-specific indicators |
| 7 | Major Conditions Strategy – Policy Framework | 2023 | DHSC | Cross-condition | As above | Policy framework | As above; distinct policy doc used in manuscript |
| 8 | One You (campaign) | 2016→ | PHE | Adults (general) | Smoking, diet, activity, alcohol, weight | Mass media + digital | High reach; not dementia-framed; no dementia outcomes |
| 9 | Couch to 5K (campaign/app) | 2016→ | NHS/PHE/Sport England | Inactive adults | Physical inactivity, weight | App + comms | High reach; no explicit brain-health link |
| 10 | Health Matters: midlife approaches to reduce dementia risk | 2016 | PHE | Health professionals | Midlife vascular risks, smoking, alcohol, weight | Evidence brief, toolkits | Good professional reach; limited public visibility |
| 11 | Reduce Your Risk (campaign) | 2019→ | Alzheimer’s Research UK | Adults | Smoking, BP, hearing, activity, alcohol, social | Public campaign | Clear dementia framing; small–moderate scale; limited long-term eval |
| 12 | Think Brain Health (campaign) | 2021→ | ARUK | Adults 40+ | Multidomain risk set | Public campaign + resources | Moderate reach; engagement high; outcome data limited |
| 13 | Think Brain Health: Check-in tool | 2021→ | ARUK | Adults (self-assessment) | Multidomain | Digital risk-check | Promising engagement; small scale; no incidence data |
| 14 | NICE NG16 – Midlife approaches to prevention | 2015→ | NICE | Adults 40–74; clinicians | Hypertension, smoking, weight, alcohol, activity | Clinical guideline | Uptake voluntary; uneven adoption |
| 15 | NHS Health Check Programme (core) | 2009→ | NHS England / Local Authorities | Adults 40–74 | Vascular risks (BP, BMI, smoking, diabetes) | Primary care check | Large reach; dementia risk rarely explicit |
| 16 | NHS Health Check – Dementia risk-reduction pilots | c. 2014–2019 | PHE/NHS | Adults 40–74 | As above + brain-health messaging | Pilots within NHS HC | Raised awareness; limited behaviour change evidence |
| 17 | All Our Health (dementia prevention module) | 2018→ | PHE → OHID | Health & care professionals | Lifestyle/vascular risks; inequalities | E-learning, toolkits | Available nationally; uptake not mandated or monitored |
| 18 | OHID Health Improvement resources (brain health relevant) | 2022→ | OHID | Professionals/system | Tobacco, alcohol, obesity, activity, CVD | Resource hub, data tools | Ongoing support; not dementia-specific |
| 19 | PHE & LSE: The economic case for prevention in dementia | 2017 | PHE/LSE | Policymakers | Multidomain; cost-effectiveness | Evidence/economic report | Synthesised econ evidence; limited operationalisation |
| 20 | NHS England Dementia Programme Update (operational) | 2024 | NHS England | NHS/system | Programme priorities; diagnosis recovery; prep for tx | Operational guidance | Emphasis on diagnosis; limited structured risk-reduction delivery |
| 21 | ARUK Public attitudes to dementia prevention | 2020 | ARUK | UK adults | Perceptions of risk & prevention | National survey informing campaigns | Informs framing; not an intervention per se |
| 22 | WHO Risk reduction of cognitive decline & dementia – Guidelines | 2019→ | WHO | Professionals | Multidomain guideline | Guideline | Cited in UK materials; not UK-specific |
| 23 | Alzheimer’s Society / sector policy resources | 2016→ | UK third sector | Public & professionals | Awareness; signposting; risk messaging | Reports, toolkits, web | Supports prevention discourse; evaluation sparse |
